# Relationships between the Toxicities of Radix Aconiti Lateralis Preparata (Fuzi) and the Toxicokinetics of Its Main Diester-Diterpenoid Alkaloids

**DOI:** 10.3390/toxins10100391

**Published:** 2018-09-26

**Authors:** Mengbi Yang, Xiaoyu Ji, Zhong Zuo

**Affiliations:** School of Pharmacy, Faculty of Medicine, The Chinese University of Hong Kong, Shatin, New Territories, Hong Kong, China; yangmengbi@cuhk.edu.hk (M.Y.); SharonChi@link.cuhk.edu.hk (X.J.)

**Keywords:** Aconiti Radix lateralis praeparata, Fuzi, diester-diterpenoid alkaloids, toxicokinetics, herb safety, herb-induced toxicity

## Abstract

The processed lateral root of *Aconitum carmichaelii Deb* (Aconiti Radix lateralis praeparata or Fuzi) is a potent traditional herbal medicine extensively used in treatment of cardiovascular diseases, rheumatism arthritis, and bronchitis in many Asian countries. Although Fuzi has promising therapeutic effects, its toxicities are frequently observed. Three main C_19_-diester-diterpenoid alkaloids (DDAs) are believed to be the principal toxins of the herb. Although toxicokinetic profiles of the toxic DDAs have already been examined in several studies, they have seldom been correlated with the toxicities of Fuzi. The current article aimed to investigate the relationship between the up-to-date toxicokinetic data of the toxic DDAs and the existing evidence of the toxic effects of Fuzi. Relationships between the cardiac toxicity and the plasma and heart concentration of DDAs in mice and rats were established. Based on our findings, clinical monitoring of the plasma concentrations of DDAs of Fuzi is recommended to prevent potential cardiac toxicities. Additionally, caution with respect to potential hepatic and renal toxicity induced by Fuzi should be exercised. In addition, further analyses focusing on the preclinical tissue distribution profile of DDAs and on the long-term toxicokinetic-toxicity correlation of DDAs are warranted for a better understanding of the toxic mechanisms and safer use of Fuzi.

## 1. Introduction

*Aconitum carmichaelii Deb* is a famous traditional Chinese medicinal herb. Its processed lateral root (Aconiti Radix lateralis praeparata or Fuzi) is extensively used in the treatments of cardiovascular diseases, rheumatism arthritis, bronchitis, pains, and hypothyroidism. In clinical practice in China, Fuzi is the constituent of more than 60 well-known traditional Chinese formulae frequently used [1]. *A. carmichaeli* belongs to the genus of *Aconitum*, which consists of over 300 species distributed in the temperate regions of the north hemisphere and 211 species in China. In total, 76 species of *Aconitum* have been used as herbal medicine or ethnomedicine in countries such as Indian, Vietnamese, Korean, Japanese, and China [1,2,3].

Although Fuzi has demonstrated promising therapeutic effects, its potential cardiotoxicity and neurotoxicity are frequently observed [2]. Thus, the clinical dosage of Fuzi is regulated in many Asian countries such as Japan, China, and Korea [4]. The clinical recommended daily dose of Fuzi is 3–15 g/person according to the Chinese Pharmacopoeia [5]. However, the actual clinical dose of Fuzi can sometimes be much higher, leading to numerous intoxication cases. From 2001 to 2010, there were about 5000 cases of aconite poisoning reported worldwide including China, Japan, Germany, and other countries [2,4,6,7,8,9,10,11,12,13]. Between 2012 and 2017 in Hong Kong, more than 41 aconite poisoning cases were reported. In Mainland China, at least 40 single or multi-person cases of fatal aconite poisoning were reported between 2003 and 2015, involving 53 victims [6]. It was found that aconite poisoning was mainly due to improper self-prescription, mistaken ingestion of *A. carmichaeli* for edible plants, and contamination of *A. carmichaeli* in other medicinal herbs [1,6,7,8]. In addition, aconite poisoning has been found in suicide and homicide cases [1,10,11,12,13].

**Figure 1:**
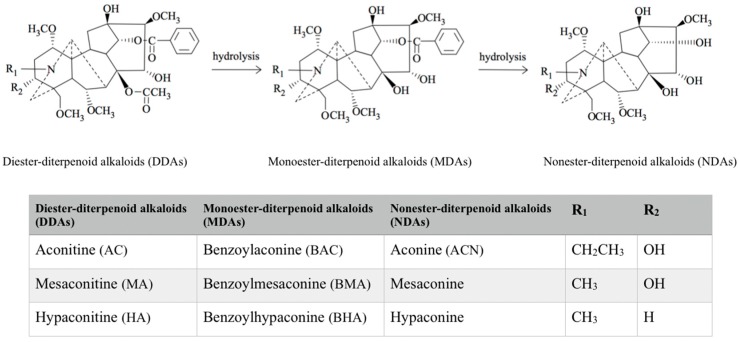
Structure of the main C_19_-diterpenoid alkaloids of Fuzi.

The principal bioactive ingredients in aconite roots are the C_19_-diterpenoid alkaloids [1,14]. Three main diester-diterpenoid alkaloids (DDAs), namely, aconitine (AC), mesaconitine (MA), and hypaconitine (HA), are believed to be the major toxic components of the herb (Figure 1) [1,14,15,16]. The toxicity of AC has been elaborated since the 1980s [1,2,14,17], whilst the toxicokinetics of the three main toxic DDAs were largely unknown until the last decade. Since the absorption, distribution, metabolism, and excretion properties of these toxic ingredients are expected to determine the safe dose of the herb, understanding of the toxicity and toxicokinetic properties of the main toxic DDAs is essential for the dosage regimen and risk control of Fuzi. The pharmacokinetics of Fuzi and their biological mechanisms have been well-delineated and reviewed [18]. Based on the previous publications on toxicity and toxicokinetics of Fuzi and its DDAs, our current review aims to investigate the toxicity–toxicokinetic relationship of Fuzi and its main toxic DDAs, which may serve as references for further clinical safety assessment. 

The following databases were searched to identify relevant literatures in both English and Chinese: Pubmed (from 1959 to August 2018) and China National Knowledge Infrastructure (from 1994 to Aug 2018). Both Latin and Chinese pinyin terms including “Fuzi”, “Lateral roots of *Aconitum carmichaelii Debx*”, “Aconite Lateralis Radix Preparata”, and “Aconite” were used as keywords to search the herb-related articles, and keywords including “Aconitum alkaloids”, “Diester-diterpenoid alkaloids”, “Aconitine”, “Mesaconitine”, and “Hypoconitine” were used for the search of compound-related articles. Any articles contained information involving the toxicity and/or toxicokinetics of DDAs in Fuzi were considered eligible for evaluation. Studies without determining the content of any DDAs in the tested Fuzi extract were excluded from the current review. 

## 2. Classification of Aconitum Alkaloids

*A. carmichaeli* comprises chemicals that range from relatively non-toxic to deadly poisonous [1]. Over 122 chemical constituents, most of which are alkaloids, have been isolated and identified from *A. carmichaelii* [1]. Based on the difference between the number of carbon atoms and the type of alkaloid structure, they can be divided into three categories: C_20_-, C_19_-, and C_18_-diterpenoid alkaloids [1,16,19]. The major ones are C_19_-diterpenoid alkaloids, which only occur the *Aconitum* species [1] and can be further divided into diester-diterpenoid alkaloids (DDAs), monoester-diterpenoid alkaloids (MDAs), and non-esterified diterpenoid alkaloids (NDAs) (Figure 1) [1]. The C_19_-diterpenoid alkaloids are also believed to be responsible for some toxicities and certain pharmacological activities of *A. carmichaelii* [1,2,18]. Three main DDAs, AC, HA, and MA as shown in Figure 1, share a common C_19_-norditerpenoid skeleton, in which the C8 and C14 position can be occupied by an acetoxy, a benzoxy, or a hydroxyl group [1,2,18]. DDAs are not stable due to the acetyl groups at C8 position and benzoyl at the C14 position, which are easily hydrolyzed in the presence of water or heat [1,2,4,20]. Upon hydrolysis, DDAs will firstly lose one molecule of acetic acid and generate corresponding MDAs, reducing the toxicity to 1/200–1/500 of that of DDAs, and further lose benzoic acid molecules at C14 position to generate corresponding NDAs with toxicity of 1/2000–1/4000 of that of DDAs [1,3]. In the Chinese medicine preparation process, the traditional way of processing Fuzi by boiling in water can reduce its DDAs content due to their hydrolysis to less toxic MDAs and non-toxic NDAs [1].

## 3. Toxicities of the Main Diester-Diterpenoid Alkaloids in Fuzi

### 3.1. Clinical Toxicities of Fuzi

According to the Chinese Pharmacopoeia, the maximal therapeutic dose of Fuzi is 15 g/person, and the maximal total content of DDAs in Fuzi is 0.01% [5], suggesting that the highest clinical recommended dose of total DDAs would be 1.5 mg/person. However, the actual clinical dose of Fuzi can sometimes be much higher, leading to a high risk of intoxication. In clinical Fuzi-intoxication cases, a combination of neurological, gastrointestinal, and cardiovascular signs and symptoms could be seen [7]. The lowest oral dose of Fuzi to induce death in humans was recorded as 0.2 g/kg. The patients may present typical symptoms, such as nausea, vomiting, dizziness, palpitations, hypotension, arrhythmia, shock, and coma [1,3] with a mean latent period of 43.6 min [2]. Electrocardiography (ECG) may show ventricular tachycardia, ventricular fibrillation, premature ventricular contractions, multifocal ventricular ectopics, sinus tachycardia, and bradycardia [1,2,3]. Death may occur from ventricular tachyarrhythmia and heart arrest, which is most likely to happen within the first 24 h after intaking *Aconitum* [1,3]. Since there is no specific therapy, cardiovascular supportive treatment is usually applied for *Aconitum* poisoning [2,3,6]. The clinical presentation of Fuzi intoxication varies depending on the dosage of Fuzi and the infirmity of patient, and no specific dose–response relationship has been reported in clinical studies so far [2,3,6].

### 3.2. Toxicities of Fuzi and Its Main Diester-Diterpenoid Alkaloids in Pre-Clinical Models

Due to limited information on Fuzi toxicity mechanisms available from clinical poisoning cases, a large number of pre-clinical studies have been conducted to investigate the toxicities and their relevant mechanisms of both Fuzi and its DDAs.

The median lethal dose (LD_50_) was commonly used as the toxicity assessment of Fuzi and its DDAs in the early years. The LD_50_ of processed Fuzi for a single oral dose was reported as 100–145 g/kg in mice. The reported LD_50_ for a single oral dose of AC was 1.0–1.8 mg/kg in mice [3,21]. For a single oral dose of MA and HA in mice, the LD_50_ values were reported to be 1.9 and 5.8 mg/kg, respectively. In contrast to those of DDAs, the toxicity of the MDAs was around 1000-fold weaker, with the LD_50_ values (single dose, oral, mice) of benzoylaconine (BAC), benzoylmesaconine (BMA), and benzoylhypaconine (BHA) as 1.50, 0.81, and 0.83 g/kg, respectively [3].

Since measurement of lethal doses could only reflect the relative toxicities of different DDAs and Fuzi, advanced methodologies have recently been applied, such as ECG, serum biomarkers, histopathology, metabolomics, and lipodomic profile changes to better characterize the Fuzi poisoning [22,23,24]. In general, damage on the cardiovascular system was obvious in almost all published reports on Fuzi-intoxicated animal models after both bolus and long-term treatment of Fuzi. Significant increases in serum creatine kinases (CKs), lactate dehydrogenase (LDH), aspartate aminotransferase (AST), and B-type natriuretic peptide (BNP) have been observed. Arrhythmias could be observed at 30–120 min post-dosing [24]. Histological changes, including inflammatory infiltration, edema, and dilated blood vessels, were observed in cardiac tissues of rodents [1,24,25]. In addition, liver and kidney damage have been reported in several toxicity studies after the single or long-term oral administration of Fuzi extract in rodents [26]. Liver damage was evidenced by the elevated alanine aminotransferase (ALT) and AST in serum, as well as by the observation of edam and necrosis in the hepatic tissue [26]. Renal toxicity was manifested as increases of serum level of creatinine and blood urea nitrogen (BUN) and histological changes of scattered lymphocytes and atrophy in renal tissue [26].

Many studies attribute the toxicities of Fuzi to the DDAs [1,14,15,16]. The three main DDAs, namely AC, MA, and HA, share the same core structure and may share similar toxic effects and mechanisms [1,2,3]. AC-induced cardiac toxicity has manifested as elevated serum CK levels, necrosis in cardiac tissue, and arrhythmias in rodents, cats, rabbits, and dogs [1,2,3,27,28,29]. Mechanisms of the cardiac toxicity of AC have been thoroughly investigated. It was found that AC could bind to the sodium channel with high affinity [30], causing the channel to activate at more negative membrane potentials, prolonging the open state of the sodium channel, and favoring the entry of Na^+^ into cytosol [2,30]. More recently, AC was found to be able to promote Ca^2+^ overload in ventricular myocytes via perturbation of the Na^+^–Ca^2+^ exchange system [2,17] and the downregulation of the sarco/endoplasmic reticulum Ca^2+^-ATPase [25], triggering apoptosis in rats [25]. Other proposed arrhythmogenic mechanisms of AC mainly focused on its alteration of the intracellular Ca^2+^ concentration [31,32,33,34,35,36,37] and were only supported by a few in vitro experiments. In addition to AC, HA was also found to induce arrhythmias in dogs and was reported to be able to induce apoptosis on an in vitro model [38], whereas reports on MA toxicity remained rare.

In summary, the cardiac, hepatic, and renal toxicities of Fuzi have been widely reported. Although the cardiac toxicity of Fuzi derived from the toxic DDAs [1,2,3] has been demonstrated in various pre-clinical observations [17,39,40,41], no study has reported the toxicities of pure DDAs on liver, kidney, or brain. Therefore, the mechanisms for Fuzi-induced liver and kidney damage could not be delineated.

## 4. Toxicokinetic Characteristics of the Main Diester-Diterpenoid Alkaloids in Fuzi

Despite the large number of studies focusing on the toxicity mechanism of Fuzi since the 1980s, the toxicokinetic profiles of the main toxic DDAs in Fuzi were not well depicted until the last decade. In this section, the toxicokinetic properties of DDAs are discussed. The absorption, distribution, metabolism, and excretion characteristics of the DDAs discovered from both clinical and pre-clinical models are summarized.

### 4.1. Toxicokinetic Profiles of the Main Diester-Diterpenoid Alkaloids in Humans after Ingestion of Fuzi

Clinical studies in *Aconitum* poisoning cases depicted some toxicokinetic characteristics of the DDAs [10,12,13]. A clinical case report determined the plasma half-life of AC in a 21-year-old man as 3 h [10]. Toxicokinetic studies on five aconitine-poisoning cases with arrhythmia showed that the elimination half-lives of AC in serum ranged from 3.7 to 17.8 h and the half-lives of MA were around 2.8–5.8 h [12]. In all five patients, the serum concentrations of AC and MA became lower than 0.05 ng/mL after 35 h and 25 h, respectively [12]. The absorption phase of the blood DDA concentration vs. time curve has never been determined in patients, so no T_max_ and C_max_ can be obtained. DDAs preferentially distribute to the liver and kidney but not to the brain. In three autopsy cases of aconite poisoning, the DDAs levels were remarkably high in the liver and kidney, relatively low in the heart and blood, and only a trace amount of DDAs was recovered in the cerebrum [13]. The concentrations of DDAs in the liver, kidney, and heart were around 2.5–22 fold, 1.8–11.7 fold, and 1–3 fold of those in blood, while the concentrations of DDAs in the cerebrum were only 3–5% of those in blood [13]. A urine sample of a patient who accidentally ingested 10 g of *A. carmichaeli* and *Aconitum kusnezoffii* was analyzed. Apart from DDAs, MDAs, and NDAs, oxidation metabolites (16-*O*-demethylaconitine and 16-*O*-demethylhypaconitine) were also present in the urine [42]. It was also found from clinical samples that the concentrations of DDAs, MDAs, and NDAs in urine were much higher than those in blood, and were continually detectable up to 7 days after overdose [2]. In addition, a higher level of DDAs could be detected in the bile than that in the serum [18,43]. These clinical findings suggest that DDAs may be eliminated via both the kidney and the liver.

### 4.2. Toxicokinetic Characteristics of the Main Diester-Diterpenoid Alkaloids of Fuzi in Pre-Clinical Models

#### 4.2.1. Absorption

Preclinical models provided more detailed toxicokinetic characteristics of the DDAs. After an oral dose of the pure compound and Fuzi extract, the bioavailability of AC was 8.24% and 4.72%, respectively [44]. Absorption of DDAs was rather fast. T_max_ of AC after ingestion of pure compounds was around 25–131 min in rats and 15–35 in mice. The permeability (P_app AtoB_) of AC, MA, and HA on the Caco-2 cell monolayer was 7.6 × 10^−6^, 8.2 × 10^−6^, and 21.5 × 10^−6^ cm/s respectively, while the permeability (P_eff_) of AC on a rat in-situ ileum perfusion model was around 0.5 × 10^−5^ cm/s. Both results from the Caco-2 cell and the in-situ intestinal perfusion model indicated medium to good absorbability of DDAs [45,46,47]. Transporters may involve and partially limit the absorption of DDAs across the intestinal epithelium. In the Caco-2 monolayer model, the efflux ratios of AC, MA, and HA were 34.6, 29.7, and 15.6, respectively, while those of the corresponding MDAs were approximately 4, and those of the corresponding NDAs equal to 1 [45,46,48,49]. Multidrug resistance protein 1 (MDR1) inhibitors, verapamil and cyclosporine A, can significantly decrease the efflux of AC on the Caco-2 cell model, and increase the intestinal permeability of AC on the rat in in-situ intestinal perfusion model [45,46,48,49]. In silico docking analyses also suggested that AC and verapamil possess similar MDR1 recognition mechanisms [46]. In addition, MK-571, an inhibitor of multidrug resistance-associated protein 2 (MRP2), exhibited inhibition on the efflux of AC but to a lesser extent than the MDR1 inhibitor on Caco-2 cells [45,48]. Taken together, MDR1 and MRP2 were involved in the transport of DDAs, partially hinder the absorbability of the toxic alkaloids.

#### 4.2.2. Distribution

AC has low protein binding (23.9–31.9%), leading to its rapid distribution to various organs [44]. The liver and the kidney are the two major organs that AC is preferably distributed to, followed by the heart, blood, the spleen, and the lung [21,49]. In the liver, kidney, and heart, AC reached a peak concentration at around 10–240 min [21,49,50]. In one study, muscle was also found as a major tissue containing AC [50]. Due to the blood–brain barrier, only a trace amount of AC was found in brain tissue [49]. Such a distribution pattern in rodents is found to be very similar to that in humans, as described in Section 4.1.

#### 4.2.3. Metabolism

It was previously hypothesized that DDAs may mainly be metabolized via hydrolysis. However, recent animal studies have revealed that the hydrolysis of DDAs to MDAs and NDAs may not be predominant in vivo [51,52,53,54]. In rats, the formation of hydrolysis metabolites (BAC and aconine) of AC was rather fast, evidenced by only a 15–30 min delay of their T_max_ in comparison to that of AC. However, their plasma and tissue concentrations were much less than that of AC [21]. At 120 min, after oral administration of pure AC, the plasma concentrations of BAC were less than 1/10 of AC, and no ACN can be detected [21], and the heart concentrations of BAC and aconine (ACN) were only 1/20–1/10 and 1/300–1/200 of that of AC [21]. Only a small amount of the hydrolyzed products (BAC) can be recovered in rabbit stomach content 4 h after oral ingestion of AC (0.5 mg/kg) in vivo [55]. It had been hypothesized that the hydrolysis of DDAs to MDAs and NDAs may also be mediated by carboxylesterase [56], but supportive evidence is lacking. In addition to hydrolyzed metabolites, 16-*O*-demethylation has been found as a common metabolic pathway of the three main DDAs via Cytochrome P450 (CYP) in both liver and intestine microsome of rats and humans [51,52,56,57]. Both 16-*O*-demethylaconitine and 16-*O*-demethylhypaconitine were detected from rats and human plasma and urine samples after oral intake of *A. carmichaeli* [42,57]. Since demethylation pathways do not alter the structure of DDAs on C8 and C14, which is responsible for its arrhythmic toxicity, 16-*O*-demethylated DDAs may also exert certain toxicity. Moreover, other metabolic pathways of the DDAs including hydroxylation, deoxylation, demethylation, didemethylation/deethylation, dehydrogenation, and ester exchange were discovered in an in vitro model using liver and intestine microsome or intestinal bacteria [51,52,56,57]. Although Phase II metabolites was not found from in vitro incubation in intestine and liver microsome, hypo-mesaconitine glucuronic acid conjugate was found in the urine of rats dosed with MA [18,56].

#### 4.2.4. Excretion

Urine is one of the major excretion routes for DDAs and their metabolites. After oral administration of AC, the urine concentration of AC peaked at 8 h post-dosing, and its major metabolite 16-O-demethylaconitine peaked at 6 h and remained detectable for up to 24 h post-dosing [2]. In addition to urine, AC can also be recovered from feces. However, the amounts of excreted DDAs and their metabolites in either urine or feces have never been compared.

#### 4.2.5. Modulation of the Transporters and Enzymes

Since DDAs have been reported to regulate the expressions and activities of some efflux transporters and drug-metabolizing enzymes, they may be able to affect the toxicokinetic profiles of themselves or other co-treated xenobiotics. DDAs and their corresponding MDAs could increase MDR1, MRP2, and breast cancer resistance protein (BCRP) expressions in cell models and mice intestine, likely via activation of the nuclear factor E2-related factor-2 (Nrf2), nuclear receptors pregnane X receptor (PXR), and constitutive androstane receptor (CAR) [47,58]. A long-term low dose of AC increased the expressions and activities of the corresponding transporters that involved in the efflux of AC, and protected against further acute AC toxicity [47]. On the other hand, the effect of the DDAs and Fuzi extracts on CYP activities and expression levels has not been systematically investigated. Treatment of AC at 0.125 mg/kg for 7 days in rats did not affect CYP3A activity or protein levels [59,60]. Treatment of HA at 2.07 mg/kg for 7 days in rats significantly inhibited CYP3A activity and induced its mRNA level, and inhibited CYP2E activity without altering its mRNA level [61]. With downregulated protein and mRNA level, CYP3A activity was inhibited by seven-day oral administration of 600 mg/kg Fuzi extract in rats [62]. Further studies are needed to systematically elucidate the effect of DDAs and Fuzi extract on the metabolic enzymes.

In summary, the ADME characteristics of DDAs from animals are similar to those found in clinical observations. Additionally, more mechanisms of the ADME processes of DDAs have been derived from pre-clinical studies, including their medium to good absorbability in the intestine, limited in vivo hydrolysis, and their modulatory effect on the activities of MDR1, MRP2, BCRP transporters, and CYP enzymes.

## 5. Relationships between Toxicities of Fuzi Extract and the Toxicokinetic Profiles of Its Main Diester-Diterpenoid Alkaloids

### 5.1. Relationship between the Cardiac Toxicity and the Toxicokinetic Profiles of the Main Diester-Diterpenoid Alkaloids after Oral Intake of Their Pure Compounds

#### 5.1.1. Relationship between the Acute Cardiac Toxicity and Toxicokinetic Profiles of the Main Diester-Diterpenoid Alkaloids after a Single Oral Dose

As shown in Table 1, the toxicokinetic and toxicity relationships on AC and HA have been studied after oral administration of their pure compounds in mice, rats, and dogs. Both the toxicity and toxicokinetic profile of AC were monitored at the same time after oral ingestion of pure AC, depicting a comprehensive profile of the dose-dependent toxic effect of AC in rats and mice. After bolus doses of 0.2 and 0.4 mg/kg AC in rats, cardiotoxic effects including decreases in heart rate and blood pressure were found to be dose-dependently aggravated along with the proportionally increased systemic exposure (AUC_0–12 h_) of AC, with no significant toxic effects observed on 0.1 mg/kg AC-treated rats [17]. Similar toxicity–toxicokinetic relationships can be found in mice. A bolus dose of 0.1 mg/kg AC in mice did not induce any observable toxicity, while 0.2 mg/kg AC induced abnormal precordial pulsation [49], and 1 mg/kg AC resulted in arrhythmia and death [21]. The cardiac toxicity of AC exacerbated as its peak concentration in heart tissue linearly increased dose-dependently. Based on these toxicokinetic data from mice and rats, the plasma concentration of AC at the lowest observable cardiac toxicity was found at around 7–13 ng/mL.

A few studies also investigated the toxicity and toxicokinetic profile of HA after bolus oral administration. After oral administration of 0.05, 0.15, and 0.45 mg/kg HA to beagle dogs, linearity of the peak plasma concentrations of HA was observed, and the cardiac toxicity (manifested as QT prolongation) was dose-dependently aggravated. Abnormality on ECG can be observed even at the lowest toxic dose of 0.05 mg/kg on beagle dogs, and the C_max_ of HA was found to be 1.53 ng/mL at such a dose [40]. The toxicokinetic profile of HA was also investigated in rats receiving a single oral dose of 0.2 mg/kg HA. Compared with AC, HA demonstrated delayed T_max_, lower C_max_, and comparable T_1/2_ [63].

#### 5.1.2. Relationship between the Sub-Chronic Toxicity and Toxicokinetic Profiles of the Aconitine after Multiple Oral Doses

Sub-chronic toxicity was induced after seven-day oral administration of 0.146 mg/kg AC to mice. Significant ventricular tachycardia, ventricular premature, and pathological changes in the myocardial tissues (including hyperchromatic nuclei and condensation of cytoplasm) were clearly observed [25]. It was also noticed that long-term exposure of a lower dose of AC might protect against the acute toxicity induced by a high bolus dose of AC. Long-term exposure to AC (0.6 mg/kg) for 14 days can reduce the mortality rate of the mice receiving a high bolus dose (1.8 mg/kg) of AC at Day 15 [47]. During a 22-day oral dose of 1 mg/kg AC in mice, the concentration of AC in blood, liver, kidney, and heart gradually decreased from Day 10 to Day 22 [21], while seven-day treatment of 0.5 mg/kg AC in rats did not alter its toxicokinetic profile significantly [44].

In summary, toxicity and toxicokinetic correlations on AC and HA after bolus doses have been established in animal models, while no such information regarding MA has ever been reported. The lowest toxic dose was found at 0.2 mg/kg for AC in mice and rats, and 0.05 mg/kg for HA in dogs, both of which are very close to the recommended clinical upper limit of the dose of DDAs in Fuzi (0.025 mg/kg in humans, equivalent to 0.15 mg/kg in rats or 0.045 mg/kg in dogs converted by the appropriate body surface area conversion factor [64]). The linear plasma kinetics of AC and HA were confirmed, and their systemic exposure levels (C_max_ or AUC) at the corresponding lowest toxic bolus dose were determined. The plasma concentration of DDAs at the lowest observable cardiac toxicity dose was found as low as 1.5 ng/mL. Therefore, risk of cardiac toxicity should be revealed when the plasma concentration of DDAs reaches such a threshold. On the other hand, toxicity and toxicokinetic correlations after long-term treatment, which are critical for the study on chronic toxicity of these compounds, have never been reported.

### 5.2. Relationship between the Heart, Liver, and Kidney Toxicities of Fuzi and the Toxicokinetics of Its Main Diester-Diterpenoid Alkaloids after Oral Intake of Its Extract

In comparison to those from the oral administration of DDAs, the toxicokinetic profiles of DDAs after ingestion of Fuzi extract are more complicated. The toxicokinetic studies of the three major DDAs after oral administration of various Fuzi extracts are summarized and correlated with their toxicities as shown in Table 2. Since the three DDAs with similar toxicity are the major toxins in Fuzi, the total amount of them from different Fuzi extract preparations was used to represent the toxicity-related dose of Fuzi for comparison of toxicities among different studies.

#### 5.2.1. Dose-Dependent Toxicities of Fuzi and the Toxicokinetic Profiles of Its Main Diester-Diterpenoid Alkaloids after Single Oral Administration

Besides the cardiac abnormity demonstrated by ECG, damage in organs including the heart, liver, and kidney were also found in animal models after a single oral dose of Fuzi extract. Only one study reported the dose-dependent toxicity of Fuzi extract with its DDA contents determined. In this study, the lowest toxic dose in rats was 2 g/kg Fuzi extract, which equals to 0.116, 0.367, and 0.586 mg/kg of AC, MA, and HA, or 1.1 mg/kg total DDAs. At such a dose, no elevated biomarker indicating heart, liver, or kidney dysfunction was observed, while mild edam was noticed in the liver and heart [26]. At a medium dose of 5 g/kg Fuzi (equivalent to 0.29, 1.168, and 1.466 mg/kg of AC, MA, and HA, or 2.9 mg/kg total DDAs), severer tissue damage to the heart, liver, and kidney was observed. At the high dose of 10 g/kg Fuzi (equivalent to 0.58, 2.336, and 2.932 mg/kg of AC, MA, and HA, or 5.8 mg/kg total DDAs), elevations of CK, LDH, AST, and urea levels were obvious, and were consistent with the remarkable damage in the heart, liver, and kidney [26]. Such toxicity investigation suggested dose-dependent damage in the heart, liver, and kidney induced by Fuzi.

On the other hand, toxicokinetic profiles of the three main DDAs after oral ingestion of Fuzi extracts have been studied in rats [68,69,70,71]. Generally, comparing the toxicokinetics after oral administration of the corresponding pure compounds, the C_max_ and AUC_0-last_ of AC and HA after administration of Fuzi extracts were significantly lower, while the T_1/2_ remained similar. The T_max_ of AC in pure-AC-treated rats was also comparable to that in Fuzi extract-treated rats. The toxicokinetic characteristics of each DDA after ingestion of mild to severe toxic doses of Fuzi extracts are discussed individually in the following paragraphs.

#### AC-Related Toxicokinetic Profiles at the Toxic Level of Fuzi

Comparing with HA and MA, the doses of AC in the Fuzi extracts were relatively low (around 0.037–0.117 mg/kg AC). In a dose range of Fuzi (equivalent to 0.46–0.66 mg/kg total DDAs, or 0.06–0.118 mg/kg AC) that refers to mild toxicity, the AUC_0-last_ and C_max_ of AC was proportionally elevated as the dosage increased, and the T_1/2_ of AC was not significantly changed between different dosage groups [44,69,70], demonstrating a linear kinetic profile at the mild toxic level. However, at a high dose of Fuzi (equivalent to 4.9 mg/kg total DDAs or 0.177 mg/kg AC), and the dose-normalized AUC_0-last_ (24.3 ± 8.9 min·kg/L) was significantly larger than those from the mild toxic dose (around 4.4 ± 0.5 to 8.3 ± 1.4 min kg/L) [71]. In addition, the lowest dose of Fuzi extract (equivalent to 0.356 mg/kg total DDAs, containing 0.037 mg/kg AC) resulted in significantly shorter T_1/2_ of AC, which may be due to the low and variable plasma concentrations [68].

#### HA-Related Toxicokinetic Profiles at the Toxic Level of Fuzi

The HA content in the Fuzi extracts was usually much higher than that of AC [72]. The doses of HA in Fuzi extracts were around 0.1–2.9 mg/kg in four different toxicokinetic studies [68,69,70,71]. Along with the increased doses, both C_max_ and AUC_0-last_ of HA were proportionally increased within the dose range from non-toxic (0.356 mg/kg total DDAs) to severely toxic (4.9 mg/kg total DDAs). The dose-normalized AUC_0-last_ values of HA were around 4.9 ± 0.9 to 8.4 ± 0.3 min·kg/L [44,69,70]. The T_1/2_ of HA (ranged from 104 ± 2 to 559 ± 62 min) varied among different studies using Fuzi at an equivalent dose of 0.46–4.9 mg/kg total DDAs [68,69,70,71].

#### MA-Related Toxicokinetic Profiles at the Toxic Level of Fuzi

When Fuzi extracts were given to the rats at low toxic doses (equivalent to 0.356–0.66 mg/kg total DDAs or 0.138–0.3 mg/kg MA), linearity was observed in the AUC_0-last_ and C_max_ of MA, with dose-normalized AUC_0-last_ of MA at around 2.8 ± 0.7 to 4.0 ± 0.8 min·kg/L, and the T_1/2_ (ranged from 251 ± 98 to 636 ± 210 min) was not significantly changed among different doses [68,69,71]. However, at a high toxic dose of Fuzi (equivalent to 4.9 mg/kg total DDAs or 1.805 mg/kg MA), the dose-normalized AUC_0-last_ (6.1 ± 1.2 min·kg/L) was slightly larger than those from the low toxic dose [71]. Additionally, the lowest dose of MA (0.017 mg/kg in the Fuzi extract) led to a much higher dose-normalized AUC_0-last_ (22.3 ± 1.8 min·kg/L), which may be due to the low and variable plasma concentrations [70].

Overall, the AUC_0-last_ and C_max_ of the three main DDAs were proportional to their doses with similar dose-normalized AUC_0-last_ values at low toxic doses (0.356–0.66 mg/kg total DDAs) of the Fuzi extract. At a high toxic dose of Fuzi extract (4.9 mg/kg total DDAs), the dose-normalized AUC_0-last_ values of AC and MA were significantly higher than those from the low dose. It is also noticed that, even under the same dose of AC and MA, their toxicokinetic parameters including AUC_0-last_ and T_max_ varied between the two groups of rats treated with different Fuzi extracts, suggesting that other co-existing components in different Fuzi extracts may affect the toxicokinetic profiles of AC and MA [69].

#### 5.2.2. Dose-Dependent Toxicity of Fuzi and the Toxicokinetic Profile of Its Main Diester-Diterpenoid Alkaloids after Long-Term Oral Administration

In clinical practice, intake of Fuzi can be at lower dose level for sub-chronic treatment that usually lasts for 1–2 weeks. Table 3 demonstrates the toxicokinetics of DDAs and toxicities of Fuzi extracts after long-term oral administration in rats. The lowest long-term oral toxic dose of Fuzi extract in rats for 15 consecutive days was found to be at 1.28 g/kg (equivalent to 0.02, 0.06, and 0.03 mg/kg of AC, MA, and HA, or 0.11 total DDAs), which is much lower than the acute toxic dose (equivalent to 1.169 mg/kg total DDAs). At such a dose, serum biomarkers indicating heart, liver, and kidney dysfunction were all elevated [23]. At a higher dosage of Fuzi extract (equivalent to 0.22 total DDAs), together with the elevations of serum markers, histological damage related to edema, inflammation, necrosis, and vascular dilatation were noticed in the liver, kidney, and heart [23]. In addition, seven-day treatment of a Fuzi extract, which did not contain any AC (equivalent to 6.3 and 4.0 mg/kg of MA and HA) in rats also led to decreased body weights and severe renal toxicity [72]. In contrary to the toxicity findings, information on the toxicokinetic profiles of the toxic DDAs after long-term oral administration of Fuzi is limited. Only one study has reported the long-term toxicokinetic profile of AC after oral administration of Fuzi extract in rats. In this study, the AUC of AC was found to be significantly increased compared with that from the single-dose group, while the T_1/2_ and C_max_ remained similar [44]. Such evidence suggests that the altered toxicokinetic profiles of the three DDAs after long-term administration of Fuzi may affect their toxicity. Further investigations are warranted to explore the relationships between long-term toxicokinetic profiles and the chronic toxicities of Fuzi and its DDAs.

In summary, a high dose of Fuzi extract usually leads to severe heart, liver, and kidney damage [26], which is evidenced by the high dose-normalized AUC_0-last_ of AC and MA after a single oral dose of Fuzi extract. Correlation between the toxicokinetics of DDAs and the toxicity of Fuzi have been preliminarily studied but are still ambiguous. To further illustrate the quantitative relationship between the toxicokinetic profile and the toxicity of Fuzi extract, two issues need to be tackled: (1) Quality variability was observed among the Fuzi extracts used in different toxicity and toxicokinetic studies. The toxicokinetics of DDAs may be affected by other co-existing components in Fuzi [69], and additive effects between different DDAs and antagonistic effects between DDAs and other components of Fuzi have been reported to modulate the toxicity of Fuzi [73]. Such quality variability of Fuzi extract leads to inconsistent relationships between toxicities and toxicokinetic profiles from different studies. (2) Due to the lack of simultaneously determined toxicity and toxicokinetic profiles of Fuzi and its DDAs, and the lack of tissue distribution data of DDAs, it is difficult to delineate the correlation between organ damage and the toxicokinetics of DDAs after oral administration of Fuzi extracts on animal models.

## 6. Conclusions

This review is the first to summarize evidence of the relationships between DDA plasma kinetics and the toxicities of Fuzi. Such relationships have been well-identified in animals receiving pure DDA compounds and have been preliminarily investigated in animals receiving different Fuzi extracts, whereas the clinical evidence of such relationships are still lacking. Based on the pre-clinical evidence, it has been demonstrated that the narrow therapeutic window and large quality variability of Fuzi extracts may significantly affect its safe use in clinical practice. Based on our findings, standardized products are essential for the safe use of Fuzi. Although correlations between the exposure of DDAs and Fuzi-induced hepatic and renal toxicities are still ambiguous, dose-dependent hepatic and renal toxicities of Fuzi have been clearly observed in pre-clinical studies. Therefore, caution with respect to potential hepatic and renal toxicity induced by Fuzi should be exercised. Since the cardiac toxicity has been well-correlated with the plasma and heart concentrations of DDAs in mice and rats, with the plasma concentration of DDAs at the lowest observable cardiac toxicity being determined, clinical monitoring of the plasma concentrations of DDAs of Fuzi is recommended to prevent potential cardiac toxicities. Further analyses focusing on the tissue concentration profile of DDAs and on the long-term toxicokinetic-toxicity correlation of DDAs are warranted to better understand the toxic mechanisms and safer use of Fuzi.

## Figures and Tables

**Table 1:** Summary of toxicokinetics and toxicity of aconitine (AC) and hypaconitine (HA) after oral administrations in animal models.

Compound	Species	Duration/Dose	Toxicokinetic Parameters				Pharmacokinetics in Heart		Toxicity Measurement				Reference
			T_max_ (min)	C_max_ (ng/mL)	T_1/2_ (min)	AUC_0-last_ (min·ng/mL)	T_max_ (min)	C_max_ (ng/g)	Serum Biomarker	Heart Histology	ECG	Behavior	
**AC**	Mice	7 days/0.146 mg/kg	NR	NR	NR	NR	NR	NR	↑ CK, AST, LDH	Hyper-chromatic nuclei, condensed cytoplasm	Ventricular tachycardia	NR	[25]
		22 days/1 mg/kg	NR	↓compared to single dose	NR	NR	NR	↓compared to single dose	NR	NR	NR	↓ Body weight ↓ Rectal temperature	[65]
		Single dose/0.1 mg/kg	23.3 ± 2.1	6.5 ± 0.5	58.8 ± 5.5	671.4 ± 63.0	10	10.6 ± 2.7	NS vs. control on CK, s-100 β	NS vs. control	NR	NS vs. control	[49]
		Single dose/0.2 mg/kg	35.0 ± 6.0	13.0 ± 0.9	69.1 ± 8.2	1578.4 ± 118.8	30	14.9 ± 2.6	NR	NR	NR	Inactive, abnormal pulsation and breathing	[49]
		Single dose/1 mg/kg	15	8.5 ± 0.4	NR	NR	14.85	71.3 ± 5.5	NR	NR	Arrhythmias	14% Dead	[21]
	Rats	7 days/0.504 mg/kg	25.0 ± 4.4	10.1 ± 2.1	211.8 ± 20.0	3329.4 ± 1199.0	NR	NR	NR	NR	NR	NR	[44]
		Single dose/0.1 mg/kg	37.5 ± 3.0	6.9 ± 0.8	NR	1485.7 ± 144.3	NR	NR	NR	NR	NS vs. control on heart rate	NR	[17]
		Single dose/0.2 mg/kg	52.2 ± 13.2	39.4 ± 3.2	100.2 ± 12.6	9043.8 ± 725.4	NR	NR	NR	NR	NR	NR	[46]
		Single dose/0.2 mg/kg	46.0 ± 15.0	9.7 ± 1.9	77.2 ± 8.4	1650.3 ± 359.2	NR	NR	NR	NR	NR	NR	[66]
		Single dose/0.2 mg/kg	25.5 ± 5.8	7.5 ± 0.8	NR	1582.9 ± 125.1	NR	NR	NR	NR	↓ Heart rate	NR	[17]
		Single dose/0.4 mg/kg	130.7 ± 10.0	7.7 ± 0.9	NR	2884.7 ± 135.9	NR	NR	NR	NR	↓ Heart rate	↓ Blood pressure	[17]
		Single dose/0.5 mg/kg	30.1 ± 9.7	8.7 ± 5.3	223.2 ± 53.0	2913.5 ± 981.1	NR	NR	NR	NR	NR	NR	[44]
		Single dose/0.5 mg/kg	47.0 ± 8.4	44.3 ± 13.0	56.1 ± 15.6	10,092.0 ± 964.8	NR	NR	NR	NR	NR	NR	[67]
**HA**	Rats	Single dose/2 mg/kg	240	2.73	145.38	834.03	15	4.6 ± 5.9	NR	NR	NR	5% dead	[63]
	Dog	Single dose/0.05 mg/kg	60	1.53	NR	NR	NR	NR	NR	NR	Prolonged QT interval	NR	[40]
		Single dose/0.15 mg/kg	60	5.74	NR	NR	NR	NR	NR	NR	Prolonged QT interval	NR	[40]
		Single dose/0.45 mg/kg	60	10.11	NR	NR	NR	NR	NR	NR	Prolonged QT interval	NR	[40]

NR: not reported; NS: no statistically significant difference; ↓: decreased; ↑: increased.

**Table 2:** Summary of toxicokinetics of DDAs and toxicity of *Aconitum L.* extract after single oral administration in beagle dogs and rats.

Species	Extract Dose	Dose of Components (mg/kg)				Targeted Compound	Toxicokinetic Parameters				Toxicity Measurements				Reference
		DDAs	AC	HA	MA		T_max_ (min)	C_max_ (ng/mL)	T_1/2_ (min)	AUC_0-last_ (min·ng/mL)	Serum	Heart	Liver	Kidney	
Beagle dog	Fuzi extract 1.5 g/kg	0.940	0.121	0.406	0.413	AC	70 ± 9	14.1 ± 0.8	273 ± 5	5514 ± 76	NR	NR	NR	NR	[74]
						HA	70 ± 9	43.2 ± 1.5	292 ± 14	18,891 ± 455	NR	NR	NR	NR	
						MA	70 ± 9	45.4 ± 1.8	375 ± 6	21,638 ± 144	NR	NR	NR	NR	
Rats	Fuzi extract 0.2 g/kg	0.356	0.037	0.181	0.138	AC	120 ± 60	1.0 ± 0.1	84 ± 16	257 ± 45	NR	NR	NR	NR	[68]
						HA	160 ± 92	5.3 ± 0.2	104 ± 2	1485 ± 243	NR	NR	NR	NR	
						MA	132 ± 96	1.9 ± 0.3	89 ± 5	477 ± 175	NR	NR	NR	NR	
	Chuanwu extract NR	0.46	0.06	0.10	0.30	AC	105 ± 16	1.3 ± 0.5	294 ± 312	353 ± 113	NR	NR	NR	NR	[69]
						HA	105 ± 18	3.0 ± 0.6	180 ± 81	786 ± 180	NR	NR	NR	NR	
						MA	105 ± 18	3.3 ± 1.0	251 ± 198	840 ± 204	NR	NR	NR	NR	
		0.66	0.06	0.30	0.30	AC	20 ± 8	2.1 ± 1.3	462 ± 292	498 ± 84	NR	NR	NR	NR	
						HA	20 ± 11	7.5 ± 3.2	310 ± 102	2184 ± 708	NR	NR	NR	NR	
						MA	25 ± 12	6.1 ± 3.8	636 ± 210	1200 ± 252	NR	NR	NR	NR	
	Fuzi extract 5.4 g/kg	2.951	0.078	2.856	0.017	AC	41 ± 14	0.9 ± 0.1	220 ± 27	340 ± 40	NR	NR	NR	NR	[70]
						HA	71 ± 14	31.7 ± 1.6	253 ± 67	13,910 ± 2504	NR	NR	NR	NR	
						MA	56 ± 8	1.0 ± 0.1	192 ± 49	380 ± 31	NR	NR	NR	NR	
	Fuzi extract 0.0384 g/kg	4.900	0.177	2.918	1.805	AC	60 ± 0	10.2 ± 1.5	644 ± 29	4297 ± 1570	NR	NR	NR	NR	[71]
						HA	60 ± 0	60.2 ± 4.3	559 ± 62	24,635 ± 100	NR	NR	NR	NR	
						MA	60 ± 0	24.8 ± 4.2	617 ± 23	10,988 ± 2192	NR	NR	NR	NR	
	Fuzi extract 4.5 g/kg	NR	0.118	NR	NR	AC	58 ± 22	3.2 ± 0.4	218 ± 86	640 ± 107	NR	NR	NR	NR	[44]
	Fuzi extract 2 g/kg	1.169	0.116	0.586	0.467	NA	NR	NR	NR	NR	NS vs. control on CK, LDH, ALT, AST, and Urea	Nuclear varies in size	Mild edema	NS vs. control	[26]
	Fuzi extract 5 g/kg	2.924	0.290	1.466	1.168	NA	NR	NR	NR	NR	↑ LDH	Dilated blood vessels	Mild edema	Scattered lymphocytes	
	Fuzi extract 10 g/kg	5.848	0.580	2.932	2.336	NA	NR	NR	NR	NR	↑ CK, LDH, AST, Urea	Nuclear varies in size	Edema	Scattered atrophy	

NA: not applicable; NR: not reported; NS: no statistically significant difference; ↑: increased.

**Table 3:** Summary of toxicokinetics of DDAs and toxicity of Fuzi extract after long-term oral administrations in rats.

Duration	Extract Dosage	Dose of Components (mg/kg)				Targeted Compound	Toxicokinetic Parameters				Toxicity Measurements				Reference
		DDAs	AC	HA	MA		T_max_ (min)	C_max_ (ng/mL)	T_1/2_ (min)	AUC_0-last_ (min·ng/mL)	Serum	Heart	Liver	Kidney	
7 days	Fuzi 4.5 g/kg	0.118	0.118	NR	NR	AC	20 ± 9	2.6 ± 1.0	384 ± 97	989 ± 67	NR	NR	NR	NR	[44]
	Fuzi 17.6 g/kg	2.066	0.000	0.804	1.262	NA	NR	NR	NR	NR	NS vs. control on BUN	NR	NR	NR	[72]
	Fuzi 35.6 g/kg	4.180	0.000	1.627	2.553	NA	NR	NR	NR	NR	NS vs. control on BUN	NR	NR	NR	
	Fuzi 88.1 g/kg	10.343	0.000	4.026	6.317	NA	NR	NR	NR	NR	↓ BUN	NR	NR	NR	
15 days	Baifupian 0.32 g/kg	0.175	0.005	0.162	0.008	NA	NR	NR	NR	NR	NS vs. control on Creatine, BUN, ALT, CK, and LDH	NR	NR	NR	[23]
	Baifupian 0.64 g/kg	0.055	0.010	0.324	0.016	NA	NR	NR	NR	NR	NS vs. control on Creatine, BUN, ALT, CK and LDH	NR	NR	NR	
	Baifupian 1.28 g/kg	0.11	0.02	0.647	0.032	NA	NR	NR	NR	NR	↑ Creatine, BUN, ALT, CK, and LDH	NR	NR	NR	
	Baifupian 2.56 g/kg	0.22	0.04	1.294	0.064	NA	NR	NR	NR	NR	↑ Creatine, BUN, ALT, CK, and LDH	Inflammatory infiltration edema	Necrosis inflammation	Vascular dilatation	

NA: not applicable; NR: not reported; NS: no statistically significant different; ↓: decrease; ↑: increase.
